# Using Supercritical Fluid Technology as a Green Alternative During the Preparation of Drug Delivery Systems

**DOI:** 10.3390/pharmaceutics11120629

**Published:** 2019-11-25

**Authors:** Paroma Chakravarty, Amin Famili, Karthik Nagapudi, Mohammad A. Al-Sayah

**Affiliations:** 1 Small Molecule Pharmaceutics, Genentech, Inc. So. San Francisco, CA 94080, USA; chakrap2@gene.com (P.C.); nagapudk@gene.com (K.N.); 2 Small Molecule Analytical Chemistry, Genentech, Inc. So. San Francisco, CA 94080, USA; famili.amin@gene.com

**Keywords:** supercritical fluids, drug delivery systems, microparticles, aerogels, membranes, foams, nanoparticles, liposomes

## Abstract

Micro- and nano-carrier formulations have been developed as drug delivery systems for active pharmaceutical ingredients (APIs) that suffer from poor physico-chemical, pharmacokinetic, and pharmacodynamic properties. Encapsulating the APIs in such systems can help improve their stability by protecting them from harsh conditions such as light, oxygen, temperature, pH, enzymes, and others. Consequently, the API’s dissolution rate and bioavailability are tremendously improved. Conventional techniques used in the production of these drug carrier formulations have several drawbacks, including thermal and chemical stability of the APIs, excessive use of organic solvents, high residual solvent levels, difficult particle size control and distributions, drug loading-related challenges, and time and energy consumption. This review illustrates how supercritical fluid (SCF) technologies can be superior in controlling the morphology of API particles and in the production of drug carriers due to SCF’s non-toxic, inert, economical, and environmentally friendly properties. The SCF’s advantages, benefits, and various preparation methods are discussed. Drug carrier formulations discussed in this review include microparticles, nanoparticles, polymeric membranes, aerogels, microporous foams, solid lipid nanoparticles, and liposomes.

## 1. Introduction

Some active pharmaceutical ingredients (APIs) suffer from poor physico-chemical, pharmacokinetic, and pharmacodynamic properties which limit their therapeutic effect. The API’s poor solubility and stability mandates frequent administration, which is highly undesired [1,2]. To overcome such limitations, micro- and nano-carrier formulations have been developed as drug delivery systems. Such formulations encapsulate the API, ideally at high drug loads, transport it to the site of action, and release it in a controlled manner; hence improving the efficacy and safety [3]. The encapsulated API is therefore protected from external conditions such as light, oxygen, temperature, pH, enzymes, and others.

There are several limitations for the conventional techniques that are currently used to manufacture drug delivery systems. For example: (1) Size reduction is a commonly used strategy to increase the dissolution rate of poorly soluble crystalline APIs or to enable delivery to the lungs. Conventional methods such as jet milling and hammer milling are regularly used to reduce particle size. These methods do not provide reproducible particle size control and also create particles with high surface energy, which in turn can lead to physical and/or chemical instability. (2) APIs are combined with polymers to make amorphous solid dispersions (ASD) to improve their bioavailability. Spray drying and hot melt extrusion are commonly used conventional techniques to make ASDs. Spray drying, while highly successful, suffers from the excessive use of organic solvents, the potential for high residual solvent in the end product, and the inability to effectively control the form of the API. Hot melt extrusion cannot be used to process thermally labile APIs. (3) In the case of sustained release applications, the API is typically embedded in either a biodegradable or non-biodegradable polymer matrix to control the release profile of the API. Many of the conventional techniques used for making these implants, like hot melt extrusion, spray drying, and solvent casting, typically cannot be applied to sensitive APIs, like proteins, peptides, and enzymes. Thus, conventional techniques used in the production of these carriers have several drawbacks, including thermal and chemical stability of the APIs, excessive use of organic solvents, high residual solvent levels, difficulty in controlling particle size and distributions, drug loading-related challenges, and time and energy consumption [4,5]. This mandates the exploration of alternative production techniques to overcome these limitations.

One of the leading alternatives is supercritical fluid (SCF) technology. Any substance can exist in its supercritical form if it is kept above its critical temperature and critical pressure [6]. The use of SCF technology has gained a lot of attention in recent decades due to the non-toxic, inert, economical, and environmentally friendly properties [7]. The physical properties of SCF, such as viscosity, density, and diffusivity, can be easily controlled by adjusting the temperature and pressure conditions [7,8,9,10,11,12]. Increasing the pressure yields an increase in the SCF’s density without significantly increasing the viscosity. An SCF can therefore be considered as a hybrid fluid sharing the best features of gases (low viscosity and high diffusivity) and liquids (high density and solvating power) [13]. A number of substances, including H_2_O, N_2_, Xe, SF_6_, N_2_O, C_2_H_4_, CHF_3_, ethylene, propylene, propane, ammonia, *n*-pentane, ethanol, and CO_2_, have been tried as SCFs, with CO_2_ being the best behaving SCF [14,15]. CO_2_ is the most widely used SCF in the pharmaceutical industry and is classified as a safe solvent by the FDA [16]. CO_2_ is an inert, colorless, odorless, non-toxic, non-flammable, cost effective, and recyclable gas. The critical temperature of CO_2_ is 31.1 °C and the critical pressure is 74 bar [17].

Table A1 briefly summarizes the recent reviews in the field to contrast them with the current review. While there have been several good reviews on SCF methodologies and the use of SCFs for specific applications in the last few years, this review gives a broad and comprehensive overview of the use of SCFs as a manufacturing technology for a variety of drug delivery systems. In this review, only a brief description of SCF-based production methods is included, with the objective of aiding subsequent discussion on drug delivery applications of SCF methods. Drug carrier formulations discussed in this review include microparticles, nanoparticles, polymeric membranes, aerogels, microporous foams, solid lipid nanoparticles, and liposomes. As the focus of this review is on drug delivery systems, the use of SCF in chromatographic and extraction techniques is not described here.

## 2. SCF-Based Manufacturing Technologies

### 2.1. Rapid Expansion of Supercritical Solutions (RESS) and Related Processes

**Figure 1:**
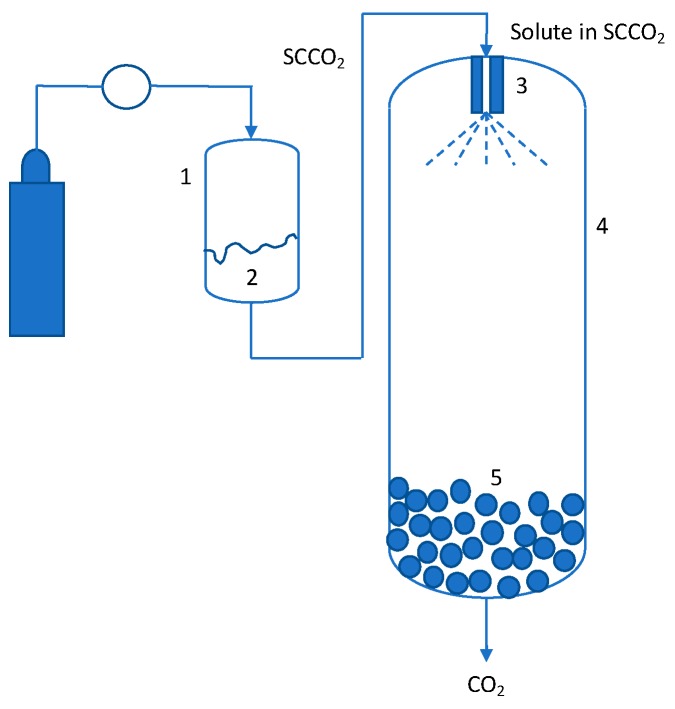
Schematic representation of the rapid expansion of supercritical solutions (RESS) process. 1—Extraction unit, 2—active pharmaceutical ingredient (API; solute) powder bed, 3—atomizing nozzle, 4—precipitation unit, and 5—size-reduced API.

Supercritical fluid CO_2_ (SCF CO_2_) can be used as a solvent for solutes that have good solubility in it. The solution is then rapidly depressurized by expanding it through a nozzle (Figure 1). This process has been termed as rapid expansion of supercritical solutions (RESS) and was first reported in the 1980s [18,19,20,21]. The depressurization leads to lower density, which in turn reduces solute solubility in CO_2_. This sudden drop in solubility causes a high degree of supersaturation (typically on the order of 10^5^ to 10^8^) of the solute, leading to its precipitation. This high degree of supersaturation leads to a high nucleation rate, resulting in a very high number of nucleation sites, thereby limiting crystal growth and producing small particles with uniform size distributions. Compared to traditional precipitation processes, RESS has the advantage of being an environmentally friendly process with mild operating conditions, where fine particles are produced in a single step without solvent residues. The size of the particles can be tuned using changes to SCF properties through pressure and temperature manipulations.

One of the main disadvantages of the RESS process is the poor solubility of solutes in SCF CO_2_. As the behavior of solutes in SCFs near the critical point is highly non-ideal, it is difficult to predict properties of the system using standard modeling approaches used for understanding fluid phase equilibrium. Thus, significant experimental work is needed to establish the useful operating range in SCF processes to produce material with desired qualities. While it is true that the solubility of a solute can increase by orders of magnitude in SCF CO_2_ compared to the ideal gas state, it will still be lower when compared to solubilities that can be achieved in liquids. Secondly, SCF CO_2_ is a non-polar solvent and, as a rule of thumb, only those compounds that dissolve well in non-polar solvents such as heptane will dissolve in SCF CO_2_. This severely limits the applicability of the RESS process to typical small molecules and biologics of interest. In order to overcome this limitation, a small amount of co-solvents and/or surfactants can be introduced at low levels to improve solubilization of the solute in SCF CO_2_. The addition of a second or third component to the SCF introduces further complexity into the phase behavior and may affect the quality of the final product, requiring further process optimization. There are further process considerations such as nozzle type and chamber design that can affect the final product quality. Extensive experimental and modeling studies have been performed to optimize particle properties for materials made using RESS [22,23,24,25].

In the last thirty years, several variations of the RESS process have been developed to expand its utility for different material types, such as polar small molecules, biologics, and polymers. These modifications include (a) rapid expansion of the supercritical solution into a liquid solvent (RESOLV) [26,27,28,29] that contains polymers, surfactants, etc.; (b) rapid expansion of the supercritical solution with an added solid co-solvent (RESS-SC) [30,31,32]; and (c) crystallization from supercritical solutions (CSS) [33,34]. Detailed discussion of these techniques is beyond the scope of this review. These techniques have been reviewed in detail elsewhere.

### 2.2. Gas Antisolvent (GAS)/Supercritical Antisolvent (SAS) and Related Processes

**Figure 2:**
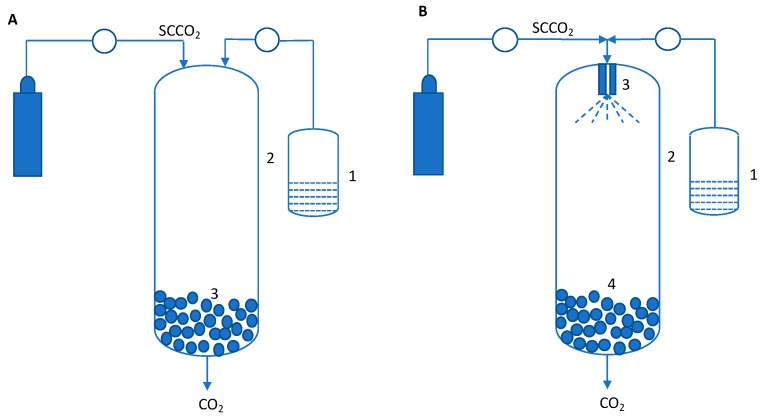
(**A**) Schematic representation of the gas antisolvent method (GAS) process. 1—Reservoir unit (API in solvent), 2—precipitation unit, and 3—size-reduced API. (**B**) General schematic of the supercritical antisolvent (SAS) process. 1—Reservoir unit (API in solvent), 2—precipitation unit, 3—atomizing nozzle, and 4—size-reduced API.

In spite of the improved solubility of solutes in SCFs, the actual solubility compared to those in typical liquid solvents is low. Thus, utilizing the SCF as an antisolvent or as a co-antisolvent significantly expands the operating space for SCF processes. This process has been referred to as the gas antisolvent method (GAS) and was first introduced in 1989 [35]. The solute (or solute + carrier) is dissolved in an SCF miscible solvent to the desired concentration. Some examples of solvents that meet this criterion are alcohols (ethanol/methanol/propanol), ketones (acetone), and dimethyl sulfoxide. This solution is then introduced into SCF CO_2_ (can also be liquid or gas), which acts as an antisolvent to help create supersaturation in the solution (Figure 2A). This process is similar to antisolvent-driven precipitations, which are commonly used separation techniques in the chemical industry. However, in contrast to a typical antisolvent process, the use of SCF CO_2_ is able to generate very high supersaturations, thereby leading to high nucleation rate, resulting in smaller particles. In addition, GAS has also shown to be useful in controlling the solid form for pure compounds and co-crystals [36,37,38,39].

It is immediately obvious that this process is more complicated than the RESS process since it involves three components. The ternary phase equilibrium needs to be carefully studied and the nucleation point well controlled in order to achieve the desired size distribution in the final product. When the incoming medium is an emulsion instead of a typical solution, the process is referred to as supercritical fluid extraction of emulsions (SFEE), wherein the SCF CO_2_ is used to extract the organic phase of the emulsion [40,41].

The GAS and SFEE processes are essentially similar, except for the feedstock. Akin to the evolution of RESS methods, GAS-based methods have also been developed to accommodate specific situations or to improve particulate characteristics such as narrower particle size distribution (PSD) and smaller sizes. In both GAS and SFEE, the feed solution is not atomized. However, if tighter control of PSD is required, atomization can be introduced and the feed solution containing the solute or mixtures of solute and polymers can be atomized using a nozzle similar to that used in the RESS process. This is referred to as supercritical antisolvent process (SAS) [42,43] and is schematically shown in Figure 2B. Several variations of SAS have been reported in the literature, based on the nature of atomization and nature of antisolvent used. For example: (a) In the SAS-EM (SAS with enhanced mass transfer) process, an ultrasonic horn is used to further make the droplets smaller after atomization [44,45]; (b) in the ASES (aerosol solvent extraction system) process, both the solvent and antisolvent are atomized [46,47]; and (c) in the AAS (atomization and antisolvent precipitation) process, precipitation is induced in the nozzle prior to atomization [48,49]. Detailed discussion of these techniques is beyond the scope of this review. These techniques have been reviewed in detail elsewhere.

## 3. Discussion

The discussion section will illustrate the use of SCFs in controlling the size, form, and shape of API particles to be used for inhalation and other routes of administration. Additionally, it will extensively review the use of SCFs in the manufacturing of drug carrier formulations, such as microparticles, nanoparticles, polymeric membranes, aerogels, microporous foams, solid lipid nanoparticles, and liposomes.

### 3.1. Active Pharmaceutical Ingredient (API) Particle Size, Shape, and Polymorphic Form

In recent years, the utilization of SCF for various pharmaceutical applications has seen a tremendous increase due to the versatility of this technology. The applications include, but are not limited to, the following: Crystallization for form control or producing novel polymorphic forms, particle size control for dissolution enhancement via size reduction (micro- and nanoparticle formation), particle engineering for specialized delivery routes such as inhalation therapy, micro- and nano-encapsulation, as well as controlled particle deposition, sterilization, coating processes, formation of solid dispersions and complexes, and generation of biopolymeric sponges or microporous foams [50]. These different applications are enabled by the role of SCF as solvent, co-solvent, solute, or co-solute. Each of these methodologies exhibit different nucleation and growth mechanisms and are selected depending on the desired product properties. Some of these pharmaceutical applications are discussed in greater detail in the following sections.

#### 3.1.1. Form Control and Polymorphism

SCF technology has been harnessed to produce novel polymorphic forms or metastable solid forms that are difficult to obtain using conventional crystallization techniques. Owing to the essential tenability of this process, form control is achieved by modification of the various process parameters, with the SCF acting as either the solvent or anti-solvent depending on the solubility of the active in the supercritical fluid (typically CO_2_). The RESS technology has been employed to obtain fine particles (mean particle size of 1.59 μm) of the metastable β-polymorph of phenylbutazone from its stable δ-form [51]. In case of tolbutamide, a hypoglycemic agent, and barbital, a sedative, the effect on SCF operating conditions (extraction pressure of 18–26 MPa, temperature of 32–80 °C and extent of supersaturation in the SCF) were found to influence the polymorphic form obtained, as well as particle size. For tolbutamide, the metastable form II or mixtures of metastable forms II and IV dominated at higher pressures (26 MPa), whereas the stable form I was obtained at a lower extraction pressure (18 MPa). For barbital, the starting form was found to be a mixture of stable form I and metastable form II which then converted to stable form I using SCF RESS at 26 MPa and 60 °C for three hours. All high pressure and temperature combinations produced metastable form II [52]. Precise control of RESS parameters can not only enable precipitation of desired polymorphs with high purity, but also offer a route to obtain new polymorphic forms that cannot be crystallized otherwise by conventional methods. Carbamazepine, existing as four anhydrous polymorphs and one dihydrate, was used as the model compound to demonstrate formation of pure polymorphic forms by changing the SCF processing parameters, where complete conversion to the stable polymorphic form III was obtained from a starting mixture of forms. This demonstrates that SCF can be used to obtain pure phases from a mixture of polymorphs without the use of any organic solvent [53]. In another example, a new polymorphic form was obtained for the excipient deoxycholine when treated under CO_2_ purge in a pressure vessel for one hour at 12 MPa and 60 °C [54].

There are also several examples of achieving form control via SCF technology where the fluid is used as a drying medium or antisolvent. Bouchard et al. used SCF as a drying technique to control the polymorphic form of glycine from solution precipitated by a direct spraying process under pressure from a co-axial nozzle. Glycine solutions of different concentrations were subjected to SCF CO_2_ (drying medium) with ethanol as the antisolvent to cause precipitation of different polymorphic forms of glycine. The authors showed that selective precipitation of either the stable α or metastable β form could be obtained by changing the solute concentration or the ethanol fraction in the system [55]. SCF is used as an antisolvent in the solution-enhanced dispersion (SEDS) technique, where the solute is poorly soluble in SCF but the base solvent shows miscibility with SCF. The principle is based on the bidirectional mass transfer of the solvent to SCF, and vice-versa. Dissolution of the solvent in the SCF causes supersaturation, which is also brought about by dilution of the SCF in the solvent, leading to density lowering and decreased solubilization. This supersaturation, augmented by solvent-SCF miscibility causes direct precipitation of the solid as dry, fine particles due to high mass transfer rates [38,56,57]. As a result, SCF provides form control for compounds showing poor solubility in SCF. It was shown in case of sulfathiazole that a change in the SCF solvent could markedly affect the polymorphic form obtained. Use of methanol led to formation of all three sulfathiazole polymorphs, whereas acetone produced only form I. The operational temperature also had a bearing on the polymorph obtained, with lower temperatures favoring forms III and IV, while higher temperature produced form I [58]. The SCF SEDS process was used to produce a new polymorphic form III with acetone at 100 bar pressure and 60 or 40 °C, and form IV with methanol at 80 °C for flunisolide (used in nasal spray to treat allergic rhinitis) [57]. Use of SEDS has been shown to be a promising approach for controlling enantiomeric purity, such as in the case of ephedrine racemates, via a diastereomeric salt formation route with mandelic acid. The resolution achieved, as determined by capillary electrophoresis, appeared to vary as a function of SCF temperature and density. The racemate was used as a starting material and crystals of >90% diastereomeric excess were obtained via a single crystallization step. These crystals showed greater purity and smoother morphology compared to those obtained by conventional crystallization techniques [59]. Oakes et al. reported exclusive pressure optimized diastereo-selectivity achieved via use of SCF drying at ~110 bar of carbon dioxide in the asymmetric catalytic sulfoxidation of cysteine and methionine. A diastereomeric purity of ~95% was obtained by this method, which could not be achieved by using conventional solvents [60].

#### 3.1.2. Particle Engineering and Micronization

Conventional techniques for micronization (such as size reduction by milling or attrition) tend to generate high surface energy in particles that may cause static charging and agglomeration, thereby leading to poor powder flow properties. In addition, shear stresses experienced during conventional micronization procedures may inadvertently lead to generation of disorder in either surface or bulk that may compromise the physical and chemical stability of the material during downstream processing or storage. Therefore, particle design technologies where size reduction procedure leads to micronized particles with minimal stress and low surface energy are highly desirable. SCF drying technology shows great promise with regard to both particle size reduction and engineering of particle properties. Unlike conventional micronization techniques with poor control over the resulting particle size distribution, SCF drying has been shown to successfully obtain micro- and nano-sized particles with a narrow size distribution. Moreover, SCF involves low temperature operating conditions, making it ideal for micronization of thermo-labile materials. The one step approach towards controlled micronization is especially useful in pulmonary delivery, wherein a tight particle size distribution is desired (1–5 μm) for drug delivery to the alveolar and bronchial tissues in the lung. For example, Turk et al. used carbon dioxide as an SCF solvent for micronization and obtained a very narrow size distribution for naphthalene (1.5–3 μm), benzoic acid (0.8–1.2 μm), and cholesterol (<0.35 μm) [25]. Unlike conventional micronization, which leads to particle agglomeration, electrostatic charging, and poor aerosolization, SCF produces free flowing micronized powders with reduced adhesion and cohesion that are more amenable to being aerosolized [61]. Rehman et al. used an enhanced dispersion SCF process to successfully produce terbutaline sulphate particles in the respirable range with improved particle deposition profile, as well as flow properties in both neat active powder and when co-mixed with lactose as compared to conventionally micronized powder samples. In addition, modification of the SCF operating conditions led to formation of different polymorphic forms with varying crystallinity and particle morphology. In another example, the glucocorticoid fluticasone-17-propionate was successfully micronized using SC carbon dioxide via the aerosol solvent extraction technique (ASES), resulting in very fine particles suitable for inhalation. Use of SCF not only enabled formulation of this anti-inflammatory active with a non-chlorofluorocarbon (CFC) propellant with comparable fine particle fraction as the CFC formulation, but also produced particles where the median size was smaller compared to jet milled powder [62]. SCF technology has been a boon for micronizing macromolecules such as proteins and genes for inhalation therapy without causing denaturation or electrostatic charging of particles [63]. In such applications, the unanimous SCF of choice is CO_2_ that acts either as the solvent or anti-solvent, in conjunction with a miscible solubilizing solvent such as DMSO or ethanol to precipitate protein solution out into powder form with the desired particle size for lung delivery. The latter principle is more commonly utilized since most proteins are not soluble in SCF CO_2_. SCF RESS falls in the former category, where SCF CO_2_ acts as the solvent for protein solubilization. A modified RESS method (RESS-N) using ethanol as a co-solvent was successfully used to microencapsulate proteins such as lysozyme and lipase in a variety of polymers, resulting in microparticles for inhalation, which showed reduced adhesive properties [64]. Considerable success has been obtained in precipitation of protein solution into powders by SAS (supercritical anti-solvent) technique, where the protein solution is dispersed at a low flow rate into the chamber with a continuous flow of SCF CO_2_, which acts as anti-solvent and reduces the protein solubility to cause precipitation. Any residual solvent is removed by the continuous flow of SCF thereafter in the reaction chamber. Yeo et al. were the first to report success with this method, where insulin dissolved in DMSO was converted into spherical and spheroidal particles (90% of the particles were <4 μm in size) with higher operating temperatures, resulting in shorter drying times and more efficient removal of residual DMSO [43]. Changes in processing conditions such as temperature and loading concentrations did not seem to have any bearing on particle morphology, and the processed insulin was found to have identical therapeutic activity as the starting material.

Besides pulmonary delivery, particle size reduction by SCF has been widely exploited to improve dissolution and, potentially, the in-vivo exposure of a poorly soluble compound for oral delivery. In one such example, particle size reduction by SCF RESS process was utilized to increase dissolution rate and significantly improve bioavailability for griseofulvin and β-Sitosterol, two compounds with poor aqueous solubility. Micronization of neat Griseofulvin produced fine particles that were 2–8 μm in size with improved dissolution, while a stable suspension of β-Sitosterol nanoparticles was obtained by rapid expansion of SCF CO_2_ charged with the active in an aqueous surfactant solution [65]. When it comes to neat API (active therapeutic ingredient), Perrut and Leboeuf have cautioned that micronization by SCF alone may not be able to improve dissolution, owing to subsequent wettability issues, changes in particle morphology, and re-agglomeration. In such cases, further processing of micronized particles, such as addition of surfactants or reformulation, may be required to fully obtain the benefits of size reduction. For example, when poorly soluble Phenacetin was micronized using RESS-SCF, a marked increase in surface area was observed for the SCF micronized particles as compared to the milled material. However, this did not translate into any appreciable difference in dissolution in pure water, owing to wettability and re-agglomeration. These issues were eliminated by including a free flow aid excipient (Aerosil) or making a 1:1 mixture of the SCF particles with mannitol [66]. SCF as antisolvent and SC carbon dioxide-aided atomization have been successfully used to produce micron and sub-micron particles of several actives, such as tartaric acid, cefonicid, tetracycline, terbutaline, and rifampicin, with controlled particle size distribution [67,68,69,70,71]. Many of these micronization studies also explored the operation variables, such as rate of carbon dioxide injection, temperature of crystallization, nozzle geometry, solute concentration, solvent to antisolvent ratio, application of sonication to atomization, and solvent choice, all of which were shown to influence the shape, size, and physical form of the dried particles [72,73].

Since SCF is an extremely tunable technology, several processing parameters are involved in influencing particle properties, such as size and morphology. Depending on whether SCF is used as a solvent (RESS) or antisolvent (GAS/gas antisolvent), these factors are: Operating temperature, pressure, residence time inside the expansion chamber, solute solubility in SCF or organic solvent, solvent to SCF ratio, rate of SCF addition, dynamics of mixing, solvent miscibility in SCF, degree of expansion of organic solvent in SCF, nozzle orifice dimensions and geometry, agglomeration during SF solution expansion, phase process route followed during expansion, or particle nucleation [74,75]. There are several literature reports exemplifying changes in particle size by modification of experimental conditions. For example, in the SEDS process where the drug, dissolved solvent, and SCF are simultaneously introduced and mixed into a reaction vessel using a co-axial nozzle, droplets and, consequently, particles of very small size (<4 μm) of Salmeterol xinafoate were produced due to increased mass transfer rate and intimate mixing between the solvent and SCF [76]. In another report, high mass transfer rates of SCF into the base solvent were attributed to be the driver for rapid nucleation and formation of smaller particles showing less agglomeration propensity [77]. In contrast, Muhrer and Mazzotti observed that although an increase in operating temperature from 19–35 °C decreased the particle size of lysozyme precipitated from a DMSO solution exposed to SCF CO_2_ via the SCF-GAS method, the agglomeration tendency of the particles increased as well [78]. Similarly, particle morphology is also affected by modifying SCF processing conditions, and a variety of particle shapes with differences in surface roughness may be obtained by varying the process parameters. For example, Yeo and Lee demonstrated that the injection rate of supercritical carbon dioxide may have a bearing on particle morphology, as seen during recrystallization of sulfamethizole solutions in acetone. Low injection rates produced large, tabular crystals, while higher injection rates led to formation of small particles that were thin and plate-like. The choice of solvent also led to changes in particle size and shape, with large platy crystals being obtained from DMF, irrespective of injection rate [72]. Using the SCF-PGSS (particles from gas-saturated solutions) process, Rodrigues et al. observed that higher expansion pressure produced a greater number of spherical particles of hydrogenated palm oil and theophylline composites [79]. In another study, Velaga et al. found out that the solvent played role in influencing particle morphology while obtaining hydrocortisone particles using SCF-SEDS. When acetone was used as the solvent, crystalline needle-like particles were obtained, irrespective of the processing conditions. However, use of methanol produced either flaky or needle-shaped, depending on the operating temperature and pressure [80]. Bristow et al. demonstrated a pressure-dependent change in particle morphology for acetaminophen, where agglomerates of spherical particles were obtained at a low pressure, while large, well-faceted crystals were produced when the pressure was raised above the critical mixture level of SCF CO_2_ and ethanol [75]. These examples amply demonstrate the flexibility provided by SCF technology in tuning particle properties for different drug delivery applications by optimizing the design space of the experiments.

#### 3.1.3. Section Summary

- SCF technology can be harnessed to produce novel polymorphic forms or metastable solid forms that are difficult to obtain using conventional crystallization techniques.
- SCF technology can be used to tailor morphology and control the polymorphic form of crystalline API in a reproducible manner.
- These methods produce free flowing micronized powders with reduced adhesion and cohesion that are more amenable to being aerosolized, making them very attractive for pulmonary delivery applications.
- SCF-based micronization is particularly useful for thermally labile materials such as recombinant proteins and antibodies.
- From a commercial standpoint, application of SCF-based micronization is more suitable for high value drug delivery applications rather than traditional oral dosage solid forms.

### 3.2. Polymeric Nano- and Micro-Particles

Polymeric nano- and micro-particle formulations have been proposed as pharmaceutical drug carriers with the potential to overcome many long-standing formulation challenges, including poor drug solubility, stability, and/or bioavailability, improving targeting to specific tissues, and modulating release behavior of the drug once administered. Polymeric particles represent a diverse class of drug carriers: They may be comprised of durable or biodegradable polymers; the drug may be molecularly dispersed within the polymer, in a separate phase or tethered to the surface; and the polymer may be a single molecule network, a homopolymer or copolymer precipitate, or a nano-structured copolymer assembly.

SCFs have been routinely employed in the production of polymeric nano- and micro-particles, and they may play one of three key roles in the process: (1) As a solvent or co-solvent to dissolve the drug, polymer, and/or other excipients; (2) as an anti-solvent to induce precipitation of the particle components; or (3) as a processing additive that contributes high solute, solvent, or co-solvent mobility, high saturation level, melting point depression, or large volumetric expansion [81]. The three main techniques for polymer particles production largely coincide with these three roles that an SCF may play. While numerous SCF-based particle production techniques have been reported [81], the following three represent the diverse contributions of an SCF to polymeric particle processing techniques: RESS, SAS, and particles from gas-saturated solutions (PGSS) where SCF is used as a high mobility additive.

In many aspects, RESS is the simplest technique, because its aim is to use an SCF as a solvent, thereby eliminating or reducing the need for organic solvents during the process. Introduction of organic solvents necessarily complicates development of any pharmaceutical formulation due to hazardous waste generation and concerns over safety and toxicity of residual solvents in the final product. However, in practice, application of RESS is relegated to a relatively small subset of applications, due to the limited solubility of polar and high molecular weight compounds in SCF CO_2_ [82]. Rapid expansion of supercritical solutions into a liquid solvent (RESOLV) is a closely related processing method that uses the same principles as RESS, but adds a solvent or solution into which the SCF is expanded. This modification is intended to limit aggregation or agglomeration of the generated particles and leads to smaller, more homogenous particles. Dalvi et al. reported a RESOLV process for encapsulation of fenofibrate in either PLGA or sodium alginate sub-micron particles, with sodium dodecyl sulfate (SDS) as the stabilizing agent [83]. PLGA and SDS were both found to be important components in preventing agglomeration of particles upon storage. Sane et al. produced poly(lactide) (PLA) particles through a RESOLV process for encapsulation of retinyl palmitate and compared Pluronic F127, Pluronic F68, and SDS as stabilizing agents [84]. Pluronic F127 produced well-dispersed, individual nanoparticles in the size range of 40–110 nm with drug loadings between 0.9–6.2%.

Since RESS and RESOLV are both limited by compound solubility in SCFs, alternative SCF-based processing techniques were developed to improve the compatibility with polar and high molecular weight components. The SAS technique employs an SCF as an anti-solvent instead of a solvent, and is one of the most widely reported SCF-based methods for production of drug-encapsulating polymeric particles [85]. In this method, a drug/polymer solution is atomized into a high-pressure chamber into which an SCF is co-injected. The SCF induces volumetric expansion of the atomized droplets, leading to supersaturation of the solution, fast nucleation, and subsequent co-precipitation of the drug/polymer solutes, which are then collected in particle form [81]. Kalani et al. reported the encapsulation of paracetamol in PLA nano- and micro-particles using DCM/acetone mixed solvents [86,87]. They reported that processing temperature and pressure were critical variables in controlling particle size, with increasing temperature increasing particle size and increasing pressure decreasing particle size. After a 7-day lag period, paracetamol was released continuously in vitro for more than 23 days with apparent zero-order kinetics. Kim et al. reported the encapsulation of the low-solubility immunosuppressive agent sirolimus in SAS-processed nanoparticles composed of polyvinylpyrrolidone (PVP) with or without various surfactants [88]. Nanoparticle encapsulation improved supersaturation and dissolution of sirolimus in vitro, and was correlated with enhanced in vivo bioavailability after oral administration in rats.

When an SCF is used as an additive instead of a solvent or antisolvent, the most common feature being exploited is the atomization enhancement contributed when it is added to a solution as a solute. PGSS is one of the mostly widely reported SCF-as-solute methods. Originally reported by Weidner et al., a mixture of polymer, drug, and other excipients in the melt is mixed with an SCF, and this mixture is then rapidly expanded through a nozzle into a lower pressure collection chamber, generating a spray of nano-droplets [89]. As this process requires polymers to be present in the melt, it would generally be required that the system be heated above the *T*_g_ of the polymer, which could result in thermal degradation of the polymer or drug. However, SCF CO_2_ is able to penetrate the polymer matrix due to its low viscosity and high diffusivity and act as a plasticizer, thereby reducing the *T*_g_ and permitting flow and atomization at lower temperatures. Key advantages of this process are that is uses a relatively small amount of SCF and, like RESS, it is solvent-free [7]. These favorable properties enabled PGSS-mediated production of human growth hormone-encapsulating PLGA/PLA microparticles at ambient temperature and without organic solvents [90]. In vitro assays demonstrated 2–3 days of continuous protein release and, critically, biological activity was not affected by encapsulation in and release from the produced particles. A similar process was employed by Perinelli et al. but using mixtures of PLA and/or PLGA and di-block copolymers of mPEG–PLA and mPEG–PLGA [91]. In vitro, formulations with high mPEG–PLA or mPEG–PLGA content produced a large burst release of 80–90% of encapsulated bovine serum albumin, followed by a short 4 days of continuous release, while formulations with lower di-block copolymer content had lower burst releases of 60–70%, followed by 20 days of continuous release. In another study, Baldino et. al. [92] described an SCF CO_2_-assisted electrohydrodynamic process to produce microparticles and microfibers of polyvynilpyrrolidone.

The application of SCFs in the preparation of polymeric particles for drug delivery, although diverse in the specific methodologies used, make use of several well-known properties of SCFs. Primarily, SCFs are used for their high compressibility, high diffusivity, high evaporation rate, and the ability to modulate their solvent power via temperature and pressure [8]. While literature reports number in the several hundred, few industrial applications have been realized for commercialized products. Further investigation may be warranted to understand where the gaps between research and industrial applications remain and how these can be closed to bring this technology to its full potential.

#### Section Summary

- SCF-based processes, including RESS, SAS, and PGSS, have been reported to permit production of drug-loaded polymeric nano- and micro-particles with distinct advantages over traditional techniques such as solvent emulsification-based methods.
- PGSS-based processes remain the most commonly used and have been reported for encapsulation of small molecules, peptides, and proteins in polymer particles via organic solvent-free processing.
- The high prevalence of these methods, as described in the literature, does not correlate with their limited use in commercial processes. To overcome this gap, significant investment and investigation are required.

### 3.3. Polymeric Membranes

In recent years, the capabilities of SCF have been expanded beyond particle engineering of actives to formulation design itself, enabling different drug delivery routes. This clean and “green” technology has been harnessed for its use as a reaction medium, thereby bypassing toxic, organic solvents. Although SCF CO_2_ is non-polar and with limited solubility of polar reactants in it, its gas- and liquid-like properties ensure high reaction rates with easy product recovery [93]. This has several applications, one of which is polymeric membrane formation. Porous polymeric membranes are heavily used in microfiltration, ultrafiltration, and dialysis, and are commonly manufactured from a polymer solution via the wet phase inversion technique, which has several drawbacks. This method uses organic solvents, the removal of which involves extensive post-processing that is both time-consuming and expensive. Additionally, this technique involves lengthy production times with little flexibility to modulate structural features of the membrane. Kho et al. successfully implemented SCF CO_2_ to induce phase separation in the polymer solution to form Nylon 6 membranes with uniform pore size with a diameter of 0.4 μm. SCF enabled rapid drying of the membrane without any collapse due to absence of liquid–vapor interface, and no additional post-treatment was needed since there were no traces of organic solvents. Moreover, the solvent dissolved in compressed CO_2_ could be recovered completely in a downstream process, away from the membrane formation chamber, using gaseous CO_2_ in a separator [94,95]. Matsuyama et al. used SCF CO_2_ as a non-solvent to induce phase separation in a polymer solution for the production of thin, flat, microporous polystyrene membranes without any structural collapse observed after reduction of chamber pressure. The effect of experimental parameters such as CO_2_ pressure, polymer concentration, as well as operating temperature on membrane porosity and pore size were investigated. The authors found that an increase in pressure, lower polymer concentrations, and higher temperatures led to enhanced pore size. The membrane porosity increased at higher operating pressures, as did the asymmetry of the membrane cross-sectional structure at higher temperatures [96]. Matsuyama et al., as well as Reverchon and Cardea, have used SCF to produce cellulose acetate membranes by using compressed CO_2_ as a non-solvent to bring about phase separation [95,97]. Matsuyama et al. investigated the role of solvent on membrane porosity and pore size, and found these membrane properties to be inversely correlated to the affinity between solvent and SCF CO_2_. Reverchon and Cardea’s research clearly demonstrated that several experimental parameters, such as CO_2_ density modulated by pressure and temperature, play a vital role in affecting membrane structure porosity and pore size. This indicates the versatility of SCF as a technology for tuning polymeric membrane properties for more widespread applications. minus the use of organic solvents and expensive treatment steps. The authors also used SCF with CO_2_ as non-solvent to produce copolymer membrane (poly(vinylidene fluoride) with hexafluoropropylene), which has a variety of biomedical applications and is also used for various filtration processes. Here too, the authors experimented with different process parameters such as pressure (80–200 bar), temperature (35–55 °C), and polymer concentration (1–20% *w*/*w*), with acetone as the solvent. The membranes so obtained were characterized by either a cellular structure with mean cell diameter of 2–6 μm or “bicontinous structures created by a sub-micrometric porous network” that existed alongside a “leaf-like” sub-morphology. This coexistence of two different kinds of structural features was attributed to the competition between liquid–liquid and liquid–solid demixing processes that is typical of such semi-crystalline polymers [98].

#### Section Summary

- Application of SCF has been demonstrated in making polymer membranes for a diverse range of materials such as nylon 6, polystyrene, and cellulose acetate. Production of porous membrane scaffolds without collapse has been demonstrated.
- SCF-based methods provide greater control of pore size and structure when compared to traditional methods using organic solvents.
- The use of SCFs can also lead to efficient downstream processing, as no additional post-treatment may be required to remove organic solvents.

### 3.4. Aerogels

Aerogels are a unique class of ultra-light, porous materials that are gaining major popularity as drug delivery systems due to their high porosity, enhanced surface area, and low density that helps to achieve high drug loadings, influence the in-vivo release kinetics of drugs, and improve bioavailability of poorly soluble compounds, as well the physical stability of the impregnated active. The theoretical basis of these drug delivery strategies can be explained using the Noyes Whitney equation:

$$\frac{dm}{dt}=A\frac{D}{h}\left(C_{s}-C_{b}\right)$$

where *dm/dt* is the dissolution rate, *A* is the surface area of the drug, *D* is the diffusion coefficient of the drug in the media, *h* is the boundary-layer thickness, *C_s_* is the concentration at the surface of the dissolving particle, and *C_b_* is the concentration of the drug in the bulk medium. Thus, increasing surface area is one method of increasing the dissolution rate of the drug. One of the methods to increase surface area is to load the drug into high surface area particles such as inorganic and organic aerogels. The drugs are impregnated into the pores of these mesoporous particles, thereby increasing the surface area of the drug.

#### 3.4.1. Preparation of Aerogels

Aerogels are typically obtained by a two-step sol–gel polymerization procedure, which involves formation of the wet gel from the solution of the network-forming material (inorganic or organic), followed by removal of solvent from the wet gel. Precursors placed in the appropriate solvent system first react to form a fine colloidal suspension, or “sol”, that further polymerizes to produce the “gel” [99]. Once formed, the gel matrix is fortified by an aging process, whereby the wet gel is introduced into an aging solution comprising of water and alcohol (typically ethanol), which helps to produce a compact gel structure that is resistant to shrinkage and damage [100]. The gel is then further subjected to a solvent exchange, resulting in an “alcogel”, where the aqueous mixture is replaced by a pure organic solvent (ethanol or acetone) to remove impurities from the pores and make it conducive for drying. Traditional drying techniques such as ambient air drying do not lead to effective removal of solvent from the aerogel network, which leads to collapse of the aerogel structure. Other specialized techniques like freeze-drying are more effective, but the slow sublimation rates needed, coupled with need for solvent exchange and expansion of solvent volume upon crystallization, make this method fraught with challenges.

On the other hand, supercritical drying avoids these problems and helps to maintain the high porosity and favorable textural properties of the wet aerogel in the dry form [101]. As a result, SCF drying is now employed as the drying method of choice, since SCF CO_2_ is a non-toxic, non-flammable, and cheap solvent that exhibits a very low surface tension at both the liquid and gas interface, and thus can be used in the drying step to obtain the aerogel without causing the network to collapse, unlike ambient air-drying conditions. Besides preserving the integrity of the aerogel network, SCF aids in formulation design by allowing modification of aerogel structural features (pore size, porosity, surface area, and density) in a controlled manner that can be judiciously used to affect both drug loading and performance of the aerogel.

**Figure 3:**
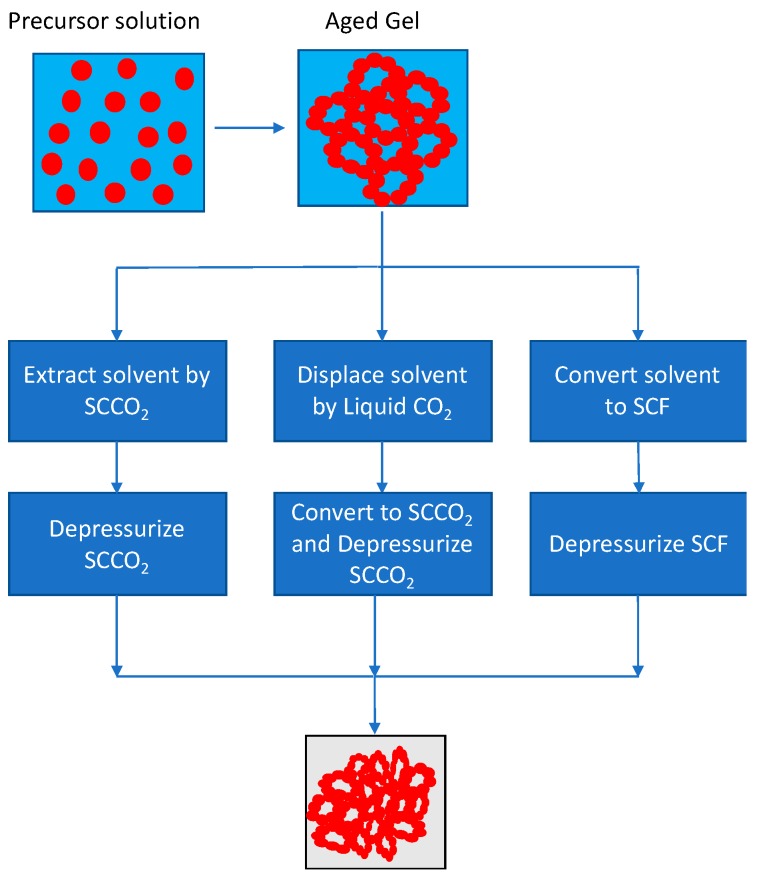
Methods used to make aerogels using SCF drying techniques.

Aerogels are generally obtained by one of three SCF drying methods (Figure 3), which are briefly described as follows [100]:

(1) Conversion of the wet aerogel liquid organic solvent to supercritical state at high temperature, followed by isothermal depressurization: In this method, the organic solvent used for aerogel preparation, such as ethanol, is heated above its critical temperature of 243 °C and pressurized above its critical pressure, following which, it is and then released to atmosphere at the elevated temperature. An obvious drawback of this method is the use of high temperature that may lead to potential degradation of the aerogel.

(2) Displacement of the aerogel solvent by liquid CO_2_, followed by its venting in the supercritical state: Here, the aerogel solvent is displaced with liquid CO_2_. The resulting solution is then pressurized above 73 atm and heated to temperatures above 31 °C (supercritical point for CO_2_). The supercritical CO_2_ pressure is then released for venting, while keeping the temperature above 31 °C.

(3) Low temperature extraction of aerogel solvent by SCF CO_2_, which acts as a drying agent: Here, instead of liquid CO_2_, supercritical CO_2_ is used to replace the aerogel solvent. The supercritical CO_2_ is then depressurized for release, while maintaining the SC temperature above 31 °C.

SCF drying can be classified into two types based on the duration of contact between the gel and the SCF CO_2_. These are as follows:

(1) Continuous or dynamic SCF drying: In the case of dynamic or continuous SCF drying, the wet gel is loaded in the autoclave or extractor, wherein it comes in contact with a continuous flow of CO_2_ maintained at supercritical temperature and pressure. This SCF CO_2_ enriched in the gel organic solvent (ethanol/acetone) is partially expanded through a restrictor (labeled V3), and this expansion leads to a drop in pressure of the SCF, leading to formation of gaseous CO_2._ The low solubilization power of CO_2_ in the gas phase leads to the stream being split to a gaseous CO_2_-rich stream and an organic-rich stream in the separator (S1). After a certain duration of time under SCF flow, when the desirable extent of pore solvent removal is achieved, the system is depressurized and the aerogel is collected [101].

(2) Batch or static SCF drying: In batch drying, the gel is subjected to an SCF CO_2_ flow for 6–12 h, following which, fresh SCF CO_2_ is introduced via the extractor to replace the one in the vessel enriched with organic solvent. This can be repeated for several cycles until the aerogel is sufficiently dried for collection [102,103].

It must be mentioned here that although SCF drying helps to produce robust aerogels at lower temperatures with high porosity and desirable mechanical properties, the solvent extraction step, i.e., removal of organic solvent from gel pore and replacement with supercritical or liquid CO_2_, is markedly slow under low temperature conditions. Depending on the final solvent content in the pores, drying of the resultant aerogel at an elevated temperature may be needed [99]. Solvent removal can be facilitated by judicious use of pore solvent that can be extracted efficiently by SCF CO_2_. Water is partially soluble in SCF CO_2_ and cannot be extracted efficiently. Thus, it is often replaced by an organic solvent during aging to facilitate solvent removal using SCF drying. Ethanol is the preferred pore liquid because of its high solubility in SCF CO_2_ and low vapor pressure, making it conducive for SCF drying [99].

There is an additional potential for further volumetric shrinkage of the aerogel prior to SCF drying during solvent exchange, where the water in the aerogel pores is replaced by a pure organic solvent such as ethanol. Stepwise solvent exchange has been demonstrated to be effective in achieving desirable low levels of volumetric shrinkage for polysaccharide and hybrid alginate-based aerogels compared to a one-step solvent immersion process [101,104]. In this method, the aerogel is soaked step-wise in different water–ethanol mixtures, with increasing concentrations of the latter at each step compared to the previous step, with the resultant solvent exchange leading to minimization of shrinkage [105]. In another example, marked reduction in gel shrinkage was observed for water–acetone solvent exchange at a low temperature of 253 K, which may be attributable to slow kinetics of mass transfer [106]. For pharmaceutical applications, ethanol is the solvent of choice compared to other solvents such as methanol, DMSO, glycerol, propylene glycol, ethylene glycol, and DMF, since they are toxic (methanol) and may cause changes in aerogel properties (such as texture, color, shape, style, strength, transparency) or lead to formation of extremely brittle aerogels (ethylene glycol) [99,107].

#### 3.4.2. Utility of Aerogels

There are an abundant number of literature reports focused on developing both inorganic and bio-based aerogels for drug delivery and biomedical applications using SCF drying. Some of these examples are discussed below, as follows:

##### Inorganic Aerogels for Drug Delivery

Most of the work to date on SCF CO_2_-based loading has been done with silica-based aerogels, as they possess good matric characteristics such as high surface area and porous structure. Caputo and co-workers studied the loading of Nimesulide into hydrophilic silica with a surface area of 800 m^2^/g and an average pore size of 20 nm [108]. The amount of Nimesulide loaded onto silica increased with increasing concentration of Nimesulide in SCF CO_2_. A Nimesulide loading of 1.53% was achieved at an operating pressure of 210 bar at 60 °C. The low Nimesulide loading was attributed to the low solubility of the drug in SCF CO_2_ and the high activation energy of adsorption. The Nimesulide-loaded silica showed much faster release in PBS when compared to the crystalline Nimesulide. The faster release was attributed to the increased surface area of the drug and the presence of Nimesulide in its amorphous form after impregnation. A variety of small molecule drugs such as Carbamazepine [109], Fenofibrate [110], Miconazole [111], Vitamin E acetate [112], Griseofulvin [113], and Ketoprofen [113] have also been incorporated into silica-based aerogels using SCF CO_2_.

Matsuyama et al. studied the loading of ibuprofen into biocompatible iron-based metal organic frameworks (MOF) [114]. Drying and impregnation of MOFs were done with supercritical CO_2_ and were compared to traditional vacuum drying. Significant improvement in surface area from 1.2 to 17-fold was observed, depending on the MOF when SCF CO_2_ drying was used. Moreover, good powdered material was obtained from SCF CO_2_ drying, while highly aggregated powder was obtained after conventional vacuum drying. SCF CO_2_ was able to preserve the pores in the MOF better than vacuum drying, and this was attributed to reduced capillary pressure due to the lack of liquid–vapor interface when using SCF CO_2_ drying. Loading of ibuprofen was done using either hexane as solvent or hexane with SCF CO_2_; 2–5 times higher loading on a weight basis was observed when SCF CO_2_-dried MOFs were impregnated with ibuprofen using hexane and SCF CO_2_. Correspondingly higher release of ibuprofen from the MOFs was observed for SCF CO_2_-based impregnation in PBS. A high drug loading of 40 *wt*% was achieved with SCF CO_2_-based drying and impregnation for the MIL-100 MOF.

##### Organic Aerogels for Drug Delivery and Other Applications

The use of bio-based aerogels with biodegradable and biocompatible polymers and polysaccharides such as gelatin, agar, cellulose, sodium alginate, chitin, and pectin has further opened up widespread possibilities for a variety of pharmaceutical and biomedical applications. Depending on the manufacturing conditions, the structural properties of aerogels, as well as their morphology (monolith, thin layers, fibers, slabs, microspheres), can be further modified, which provides the much needed flexibility for drug delivery and in vivo release kinetics [115,116,117]. For example, Martins et al. manufactured hybrid starch–alginate aerogels using SCF technology with improved mechanical properties and an increase in macroporosity, with pore size being >100 μm. In vivo tests showed them to be non-cytotoxic, bioactive, and effective scaffolds for cells to adhere to and proliferate from [116]. Hybrid chitosan-based hydrogels, such as those modified with anionic polyelectrolytes, may also be applied to transdermal, oral, and ophthalmic drug delivery, as well as tissue engineering [118]. Hile et al. investigated the encapsulation of basic fibroblast growth factor (bFGF) in PLGA scaffolds with SCF CO_2_ for use in tissue engineering [119]. Initially, an emulsion containing bFGF, BSA, and PLGA was made in methylene chloride and water using sonication. Then, the emulsion was converted into a porous bFGF-containing matrix using SCF CO_2_. In this work, the authors compared SCF CO_2_-based encapsulation against the salt leaching technique. The total amount of protein released was found to be higher for the 65/35 PLGA polymer when compared to the 80/20 polymer, which is in line with increased hydrophobicity of the 80/20 PLGA. The protein release from the 65/35 PLGA polymer made using the salt leaching technique was found to be lower by a factor of 2 when compared to 65/35 PLGA polymer made using SCF CO_2_. However, the amount of active protein released was an order of magnitude lower from the SCF CO_2_-based scaffolds when compared to the scaffolds made using salt leaching. These findings demonstrate that the bFGF was likely deactivated during processing with SCF CO_2_. While high amounts of protein encapsulation are possible, the amount of active protein is affected by the process. The amount of residual methylene chloride was also found to be higher in the SCF CO_2_-based PLGA scaffolds. This study demonstrates the issues associated with encapsulating delicate materials like proteins using SCF CO_2_.

In comparison to silica-based material, organic aerogels have lower surface area. Nevertheless, their use as porous carriers for drugs is increasing based on their GRAS status and their better biocompatibility and/or biodegradability. Cabezas and coworkers demonstrated the loading of 5-Fluorouracil into porous biodegradable PLA and PLGA scaffolds [120]. Initially, the polymers were foamed using SCF CO_2_ via a pressure quench method. In this technique, the *T*_g_ of the polymers is depressed due to absorption of SCF CO_2_. Depressurization leads to escape of CO_2_, which in turn nucleates bubbles in the polymer matrix, resulting in a formed structure. In a single step, 5-Fluorouracil was loaded into PLA and PLGA. The same group also studied the incorporation of indomethacin in PLA/PLGA matrix [121]. Yoganathan et al. studied the loading of ibuprofen into polycaprolactone [122]. The objective of this study was to create sustained release matrices. By varying the temperature and pressure of SCF CO_2_, they were able to vary the drug loading from 1.8 to 27.2%. Interestingly, the drug loading did not match solubility of ibuprofen in SCF CO_2_ under the conditions investigated. Thus, the drug loading is dependent on a complex interplay of factors such as solubility, partitioning of drug between the SCF CO_2_ phase and the polymer, and ability of the drug to penetrate the polymer. Free ibuprofen showed complete dissolution in DI water within 5 min. In contrast, PCL loaded with 27.2% ibuprofen showed sustained release, with only 30% ibuprofen released in 72 h. In another example of drug-loaded aerogels enabling sustained release of API, whey protein-based aerogels were prepared using SCF technology that allowed the gel nanostructure to be preserved. This aerogel showed good mechanical strength, high BET surface area, high drug loading capacity, and a pH-controlled swelling behavior upon rehydration that allowed for the controlled release of the loaded API (ketoprofen) at pH 1.2 and 6.8 in in vivo tests [123]. In another study, Marin et al. studied loading of ibuprofen into silk fibroin aerogels [124]. Initially, silk hydrogel was first produced from aqueous solution of silk saturated with SCF CO_2_ at 100 bar and 40 °C. The hydrogel was then soaked in solutions of increasing ethanol concentration and, finally, pure ethanol. The ethanol was then removed using SCF CO_2_ at 100 bar and 40 °C to produce silk fibroin aerogels. The silk fibroin aerogels had a surface area of 424 m^2^/g and a density of 0.058 g/mL. Ibuprofen was then loaded into the aerogel using a third SCF CO_2_ process. A total loading of 20.6% by weight was achieved. In in vitro release studies in PBS, free ibuprofen released completely by 15 min, whereas silk aerogel released the drug over a 6 h period. Thus, sustained release was demonstrated using SCF CO_2_-based loading with silk fibroin. The production of polysaccharide-based aerogels follows a procedure similar to that outlined for silk fibroin aerogel. The production and the use of polysaccharide-based aerogels for drug encapsulation have been reviewed elsewhere [101]. As an example, Mehling et al. studied the encapsulation of ibuprofen and paracetamol in various polysaccharide matrices [125]. They found that one could tailor the structural features of the matrix to change drug release profile.

Bio-based aerogels have also been prepared as microspheres for drug delivery using SCF technology, since they are preferred over other aerogel morphologies (monoliths, powders, and granules) due to improved flowability, ease of handling, reproducibility in processing, and reduced inflammatory response triggering as a result of the absence of sharp edges in the delivery system [126]. Garcia-Gonzalez et al. used polysaccharide- based (starch, pectin, alginate) microspheres to harness the advantage of polysaccharide biocompatibility and drug loading ability of dry aerogels as delivery systems for poorly water soluble drugs such as ketoprofen and benzoic acid. The aerogel microspheres were produced using the emulsion–gelation method, followed by SCF drying, and the active was loaded onto the aerogels by supercritical impregnation, where the substrate was exposed to SCF CO_2_ carrying the solubilized actives. The drug loading efficiency (ranging from 11–24 *wt*%) of the aerogel was found to be dependent on several factors, such as drug type, aerogel material type, drug–aerogel carrier interaction, aerogel surface chemistry, and specific surface area. The impregnated drug was characterized to be amorphous with good physico-chemical stability upon storage. The authors also showed the aerogel erosion mechanism (in contact with aqueous medium) and release profile of the drug to be dependent on the matrix type, with pectin and alginate aerogels showing fast erosion and starch aerogel matrix displaying a slower, more gradual erosion. Ketoprofen release from all three polysaccharide-based aerogel microspheres was similar in pH 6.8 buffer solution, with high dissolution rate-driven immediate release of ketoprofen in the first 2 h, followed by a more sustained release type profile until a plateau condition was reached. Overall, pectin- and alginate-based microspheres allowed about 80% of the API to be released in 2 h as compared to only 54% being released by starch aerogel microspheres. In addition, alginate microspheres were found to significantly enhance ketoprofen dissolution by accelerated drug release at simulated gastric pH (pH 1.2) conditions. This study demonstrated that the choice of polysaccharide type clearly influenced the release profile of poorly water soluble APIs, which makes these aerogel microspheres even more lucrative for drug delivery [126].

##### Specialized Aerogels

In addition to biocompatible inorganic and biodegradable organic aerogels, more complex ones such as surface functionalized aerogels, composite aerogels, and layered aerogels are also being explored for drug delivery [127]. In surface-functionalized aerogels, the fast matrix erosion of hydrophilic surfaces in water or aqueous media is stymied by including hydrophobic groups [128,129]. This surface modification inhibits the intrusion of the dissolution medium within the aerogel matrix, and therefore alters the drug release profile to be more controlled or sustained, via drug diffusion through the aerogel pores. Moreover, the drug release profile can be further regulated by changing the surface hydrophobicity, as well as density, of such aerogels. Surface modification is typically introduced during the supercritical drying step, since exposure of the aerogel to a solution of surface modifiers can jeopardize its integrity [130]. Composite aerogels are those where secondary materials are used to diversify the functionalities of the aerogel matrix [131,132,133,134,135,136,137]. The idea is to include diverse materials with high surface areas and with different surface properties to maximize drug loading into aerogels. For example, inclusion of polymers as secondary materials in aerogels may not only improve the mechanical strength of the aerogel, but may also provide controlled release of the impregnated active and eliminate the need for supercritical drying if the matrix is robust enough to withstand air drying. Finally, multilayered aerogels have also been found to be attractive drug delivery systems with several advantages, such as (a) providing a protective layer to hydrophilic aerogels to prevent their collapse in aqueous media, (b) regulating drug release from the core matrix, (c) sequential release of different drugs from different layers depending on their hydrophobicity, and (d) stimuli-sensitive release for targeted delivery, where different drugs are released under different physiological conditions [138,139,140].

#### 3.4.3. Section Summary

- Aerogels are a unique class of ultra-light porous materials used as drug delivery systems, owing to their high porosity, increased surface area, and low density that helps to achieve high drug loadings, influence the in vivo release kinetics of drugs, and improve bioavailability of poorly soluble compounds and the physical stability of the loaded active.
- Aerogels are produced via a two-step method of sol–gel polymerization, followed by a drying step involving the removal of the pore solvent to produce a rigid gel. Supercritical fluid drying is the most effective drying method to produce mechanically stable aerogels that do no collapse and retain their porosity and texture.
- Both organic and inorganic aerogels are extensively used for drug delivery, the latter being preferred due to biocompatibility and biodegradability. Inorganic aerogels are mainly silica-based, whereas bio-based aerogels are made in conjunction with organic polymers such as chitin, cellulose, etc. Specialized aerogels such as functionalized, composite, and layered aerogels are also being used to render additional physico-chemical attributes to modulate drug delivery further.

### 3.5. Microporous Foams

Supercritical foaming is one of the many diverse applications of SCF technology that is used in producing three-dimensional (3D) drug-releasing microporous polymeric foams, which have a wide variety of biomedical applications such as drug delivery devices for controlled release of actives, implant drug delivery for chemotherapy, carrier agents for DNA delivery, and tissue engineering scaffolds to promote cell proliferation [50]. The high porosity, low density, biodegradability, and biocompatibility of naturally occurring polymers or those derived from biodegradable materials make them ideal as in vivo structural materials [141,142]. For example, Trujillo de-Santiago et al. used supercritical foaming to produce foams out of thermos-plasticized maize-derived materials such as zein (maize protein), which acted as scaffolds in cell culture experiments, wherein mouse fibroblasts and two separate prostate cancer cell lines were found to attach to the foam and proliferate. In another example, Tayton et al. explored two bio-based polymers as substitutes for the more expensive and less effective allografts in impaction bone grafting (IBG), owing to their biocompatibility and suitable mechanical properties [143]. The scaffolds of poly(d,l-lactide) and poly(d,l-lactide-*co*-glycolide) were prepared by melt processing, followed by SCF CO_2_ foaming. The pore sizes of the foams ranged from 50–200 μm, and these porous scaffolds were found to have superior shear strength compared to allografts. Cellular studies also showed enhanced cell numbers, viability, and increased osteogenic differentiation on porous polymer scaffolds compared to non-porous ones. Overall, the porous polymer scaffolds showed markedly improved cohesion and cellular compatibility compared to the block, non-porous polymers without any marked loss in mechanical strength, and thus appeared to be promising candidates for IBG. In an example of use of polymeric foams as drug carriers, microporous PLGA foams impregnated with paclitaxel were obtained using a two-step process of spray drying and supercritical foaming. SCF foaming reduced the residual solvent content in the polymer due to high miscibility of SCF CO_2_, with the organic solvent used in the spray drying step. These disks were tested to potentially act as surgical implants for achieving a sustained release of the active. In vivo release tests showed a constant release rate of the drug for up to 8 weeks, and in vivo intracranial implants in mice showed maintenance of therapeutic concentrations in the brain, even after 28 days of implantation [144]. There are several other literature examples of foams encapsulating actives such as curcumin, chitosan, and gentamicin that are used as scaffolds, where the drug is released either by diffusion or upon degradation of the polymer matrix [145,146,147].

**Figure 4:**
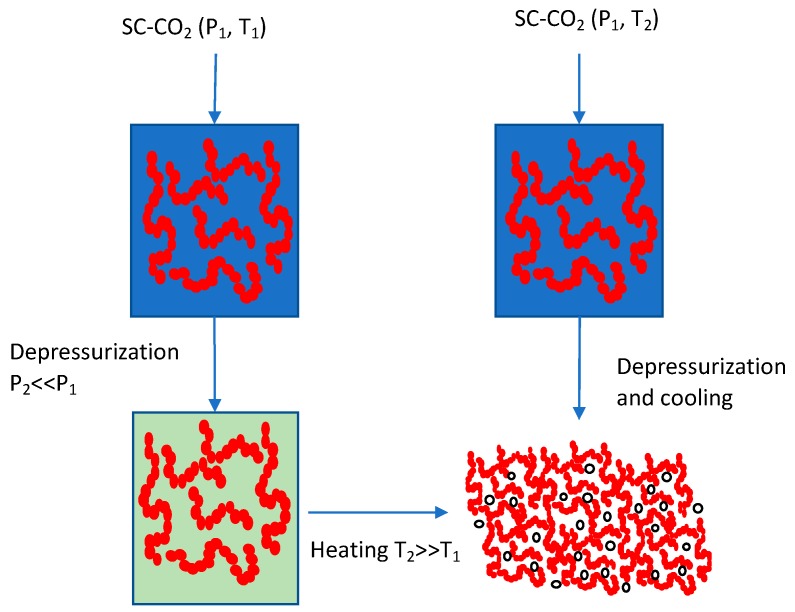
Schematic representation of SCF-based methods to make polymer foams. Left: Introduction of SCF CO_2_ at a pressure *P*_1_ and temperature *T*_1_ (<*T*_g_), followed by depressurization and heating to remove trapped CO_2_. Right: Introduction of SCF CO_2_ at a pressure *P*_1_ and temperature *T*_2_ (>*T*_g_), followed by simultaneous depressurization and cooling to remove SCF CO_2_. o: Represents bubbles in the polymer foam.

Production of microporous scaffolds using SCF technology is a complex and dynamic process which involves two basic steps: (a) Pressure induced sorption/dissolution of SCF in the polymer matrix to form a polymer/gas “solution”, followed by (b) bubble nucleation and growth brought about by reduction of pressure or increase in temperature. The bubble nucleation and growth are caused by lowering the solubility of the SCF in the polymer matrix by depressurization, as a result of which, the oversaturated SCF bubbles within the polymer matrix to create these porous morphologies, which are either closed cellular or open pore in nature. Foaming stops due to polymer vitrification when the glass transition temperature of the polymer–SCF mixture exceeds that of the system temperature. Figure 4 is a schematic representation showing foam production [142].

Nucleation and, subsequently, bubble growth are critical to the foaming process and are affected by several factors, such as viscosity and density of polymer/CO_2_, diffusivity of CO_2_ in the mixture, and relaxation time for the polymer swollen by SCF CO_2_ [148]. For example, bubble growth near the surface may lead to surface blisters that may rupture if the melt viscosity of the polymer melt with entrapped CO_2_ is not sufficiently high [149]. Low viscosity of the melt may lead to production of dense skin layers caused by rapid diffusion of SCF (typically CO_2_) from the surface that leads to an increase in the glass transition and modulus of the system. When polymer blends are used for manufacturing foams, the composition of the blend and the interaction of its components with CO_2_ affects the mode of bubble nucleation across different domains. In general, conditions that facilitate a greater amount of CO_2_ dissolution and high melt viscosity produce stable foams [150,151]. Finally, the crystallinity of the polymer, affected by foaming conditions, may also affect foam morphology. For example, in block copolymers of poly(methyl methacrylate) (PMMA) and polystyrene (PS), if decompression pressures render PMMA rubbery and PS glassy, the bubble growth will be restricted by the glassy polymer matrix [152].

Although CO_2_ is usually the SCF of choice, sometimes nitrogen is used in conjunction with CO_2_ for SCF foaming. Factors critical to producing stable foam include supercritical gas concentration, temperature and pressure at which bubble nucleation is induced, mode of pressure reduction, and cooling program after foaming is complete [142,153]. The final product properties (porosity, pore size, density, inner morphology, pore size distribution) are dependent on several process conditions, such as (a) gas type (CO_2_ or N_2_), (b) gas concentration in the polymer matrix (the upper limit being dictated by its solubility), (c) diffusivity of SC gas in the matrix, (d) interfacial tension between the polymer/gas solution and SC gas, and (e) effect of sorption and decompression stage on the polymer transition temperatures and the associated processes (melting, crystallization, vitrification, plasticization), as well as its rheological properties (viscosity and modulus) [153,154,155,156,157,158,159]. In general, at a given temperature, faster decompression leads to smaller pore sizes, while foaming from higher temperatures leads to increase in pore size for a given gas concentration and rate of pressure drop [160]. The polymer type and properties that are key to being conducive to the SCF foaming technique are as follows: (a) Polymer crystallinity (amorphous ones such as polystyrene are preferred over semi-crystalline polymers), (b) ability of the polymer to reasonably solubilize the gas in its matrix, (c) sufficiently high melt strength, (d) ability to crystallize or vitrify near the processing window, and (e) viscoelastic properties of the polymer matrix in the presence of dissolved SCF to enable formation of a stable, microporous foam.

Batch foaming, extrusion foaming, and foam injection molding are the three common techniques utilized in SCF foaming to produce polymeric foams [142]. In batch foaming, the polymer sample is exposed to CO_2_ in a high-pressure vessel to facilitate sorption, followed by decompression that allows the bubbles to be nucleated. For low operating temperatures, if the polymer is too rigid to produce foam upon pressure release, it is removed from the vessel as a solid matrix with entrapped CO_2_ and then heated for softening that induces bubble nucleation and foaming. This particular foaming process is known as “solid-state foaming”. Although batch foaming is routinely used in research and for small amounts of sample, its low productivity is what bars its use in industry settings, where extrusion foaming is the technique of choice. Here, CO_2_ is introduced into an extrusion barrel and mixed with the molten polymer under high pressure. At the nozzle exit of the barrel, this polymer–CO_2_ solution experiences a pressure drop and undergoes de-mixing, which leads to bubble nucleation. Here, CO_2_ is used as a plasticizer that allows the operational temperature to be lowered, and helps in modification of the rheological properties of the melt that acts as the expansion agent as the polymer exits the nozzle [150,161,162]. Although extrusion foaming is preferred for continuous manufacturing and scale-up, there are several limitations associated with this method. These are: (a) Limits to pressure levels achievable at the CO_2_ charge point as imposed by the extruder barrel/vessel, (b) time available for CO_2_–polymer mixing before the latter exits from the barrel nozzle, and (c) the melt viscosity required to prevent the CO_2_ from escaping (which leads to foam collapse) upon exiting the vessel. The third alternative is to use foam injection molding that is both cost effective and a proven method to obtain foams with intricate 3D geometries. Here, the mold cavity is first filled with the polymer, and then a part of the mold is removed along its thickness after a certain period of time. This in effect leads to a “die expansion”, which causes the dissolved CO_2_ in the polymer to experience depressurization that leads to uniform foaming along the entire injection mold [142].

For polymeric foams encapsulating active therapeutic agents for drug delivery, either a single-step or two-step encapsulation and foaming technique is employed for drug loading in the foam. In the single-step process, the SCF (typically SCF CO_2_) acts as both the solvent for the active, as well as the solute diffusing the polymer matrix. Although the advantages are low processing temperature for thermolabile drugs, the main drawback is that the extent of drug loading in the polymeric foam is limited by the solubility of the active in SCF CO_2_. The two step process is thus used to achieve higher drug loading, improve encapsulation efficiency, and enable a wide variety of actives (both hydrophobic and hydrophilic) to be encapsulated in the foams. In this two-step encapsulation and foaming process, a drug-loaded polymer matrix is first obtained by a separate technique, such as solvent casting or spray drying or emulsification, which is then converted to a microporous structure via SCF foaming, where the drug remains encapsulated in the porous matrix. Although an organic solvent is used in the first step, the high miscibility of SCF CO_2_ in the solvent ensures its near complete removal from the final product, thereby significantly reducing the residual solvent content in the foam [50].

#### Section Summary

- Supercritical foaming is one of the many diverse applications of SCF technology that is used in producing three-dimensional drug-releasing microporous polymeric foams, which have a wide variety of biomedical applications.
- Nucleation and bubble growth are key to foaming and are affected by several processing conditions, as well as physico-chemical and viscoelastic properties of the polymer melt and SCF. CO_2_ is the SCF of choice for producing polymeric foams.
- Batch, extrusion, and foam injection molding are used for manufacturing of microporous foams.
- Polymeric foams produced by SCF technology are used for drug delivery of both hydrophilic and hydrophobic drugs that can be encapsulated in these foams.

### 3.6. Solid Lipid Nanoparticles

Solid lipid nanoparticles (SLNs) are sub-micron-sized colloidal drug carriers composed of a lipid matrix encapsulating a drug and/or other cargo, typically dispersed in an aqueous phase with or without a surfactant. While similar in size to other sub-micron-sized colloidal drug carriers, such as liposomes, micelles, nanoemulsions, and polymeric nanoparticles, SLNs are distinguished by their physicochemical structure, defined by a continuous lipid matrix that exists as a solid at ambient temperatures. This solid lipid matrix affords an excellent formulation opportunity for delivery of medium-to-low-solubility compounds, which remains a persistent challenge for many pharmaceutical compounds. The original motivations for development of SLNs continue to inform their investigation: High biocompatibility stemming from the use of physiological lipids, enhanced delivery of poorly soluble drugs due to the lipophilic core, enhanced stability of the encapsulated drug due to the solid matrix, potential for controlled release of encapsulated drugs based on partitioning out of the lipid matrix, and ease of production using scalable processes [163].

Production of SLNs is routinely carried out by traditional dispersion techniques, taking advantage of the melting properties of the lipid or its solubility in non-aqueous solvents. In the hot homogenization technique, a melt consisting of the lipid and drug to be incorporated is prepared and dispersed in an aqueous solution containing a surfactant above the melting temperature of the lipid. This emulsion solution is then homogenized at high pressure, followed by cooling of the suspension, thereby solidifying the lipid core and allowing for re-crystallization. In the emulsion precipitation technique, a solution of lipid and drug in a water-immiscible solvent is emulsified in an aqueous solution containing a surfactant, followed by removal of the solvent by evaporation, extraction, or dilution, and subsequent precipitation of the lipid–drug mixture. The mechanism of SLN formation, as well as a multitude of process parameters, play a defining role in the structure and physicochemical properties of the resulting particles. These may be driven by: (a) Kinetics of nucleation, precipitation, crystallization, and/or solvent extraction; (b) properties and dynamics of surfactants in the aqueous phase; and (c) solubility of the drug in the lipid core before, during, and after solidification [40].

The advantage of SCF technology as it relates to preparation of SLNs is that relatively small pressure changes in an SCF can yield a significant change in the solubility of a substance in the liquid. In a typical applied process, controlled depressurization of an SCF can rapidly create supersaturation of dissolved compounds and their subsequent precipitation. This phenomenon can be used in the preparation of SLNs in various forms, including SFEE, PGSS, RESS, and GAS processes.

In 2007, Chattopadhyay et al. reported the application of SCF technology to the preparation of SLNs by the SFEE technique [40]. This process involves formation of a homogenized oil-in-water emulsion comprised of lipid (in this case tripalmitin, tristearin, or Gelucire 50/13) and drug (indomethacin or ketoprofen) dissolved in chloroform with a surfactant (soy lecithin) dispersed in an aqueous phase of sodium glycocholate, which is then introduced into an extraction column with counter-current flow of SCF CO_2_. Within the extraction column, SCF CO_2_ is simultaneously extracting solvent from the oil phase of the emulsion and expanding the organic phase of the emulsion, ultimately resulting in precipitation of the lipid–drug mixture in an aqueous nanodispersion. Noted advantages of this process over non-SCF-based solvent extraction methods are higher solvent extraction efficiency, more uniform particle size distribution, depression of the lipid melting point, and plasticization of the amorphous lipid structure.

A similar report employing an SFEE process by Shekunov et al. in 2006 sought to elucidate the process parameters responsible for controlling the resulting SLN size, morphology, crystallinity, residual solvent, and dissolution characteristics [41]. For the model systems under investigation, it was found that particle size was largely dictated by emulsion droplet size, drug concentration, solvent content, and rate of solvent extraction. Residual solvent levels in the low parts per million range were achieved, SLNs displayed high levels of crystallinity, and dissolution rates were 5-to-10-fold faster than micronized powders. This paper neatly addressed the benefits of SCF-mediated SLN production and the critical-to-quality parameters that should be investigated in designing such a process.

PGSS has garnered significant attention in the production of SLNs, owing to its simple process, solvent-free nature, and direct production of dry SLN powders. In the PGSS process, lipid and drug are melted and then saturated with SCF CO_2_, leading to the formation of a gas-saturated solution. This solution is then passed through a nozzle, resulting in rapid depressurization and precipitation of the lipid–drug matrix, and dry particles are collected in the depressurization chamber. PGSS has been found to be an attractive choice for sensitive molecules, including peptides and proteins [164], since it can be operated solvent-free and requires relatively mild conditions in the initial melt step due to the plasticizing effect of melting lipid and drug under an SCF [165,166].

Application of PGSS has been reported by several groups, including Rodrigues et el. who used hydrogenated palm oil for encapsulation of theophylline [79], Wang et al. who encapsulated ibuprofen in myristic acid and tripalmitin [167], Pestieau et al. who used Gelucire 50/13 to encapsulate fenofibrate [168], and Sao Pedro et al. who produced curcumin-loaded tristearin/soy phosphatidylcholine particles using a small amount of organic solvents during the process [169]. Solid lipid particles produced in these reports were all in the >1000 nm range, indicating a process limitation of PGSS for lipid particles.

In order to overcome this limitation and produce sub-micron solid lipid nanoparticles, the gas-assisted melting atomization (GAMA) process was developed. In this technique, lipid and protein are mixed in the melt under SCF CO_2_. The contents of this chamber are then atomized into a precipitation vessel with a coaxial air stream, where rapid depressurization of the mixture results in supersaturation and precipitation of the lipid–drug mixture. The GAMA process has been used to produce protein-loaded SLNs due to its relatively mild operating conditions. Salmaso et al. encapsulated insulin [170] and recombinant human growth hormone [171] in tristearin/phosphatidylcholine/PEG mixtures, resulting in spherical particles in the 80–400 nm size range, and both proteins were released under physiological conditions in their active form for up to 100 h.

In another alternative process termed RESS, an SCF is used as a solvent to dissolve the lipid and drug. This solution is then depressurized through a nozzle into a low-pressure expansion chamber, causing the SCF solution to become supersaturated and, consequently, precipitation of the lipid–drug matrix. This process was employed for the production of ibuprofen-loaded stearic acid SLNs [172] and carbamazepine-loaded stearic acid SLNs [173]. A limitation of the RESS process is the use of SCF as the solvent for dissolution of the lipid and drug. In cases where the lipid or drug are not adequately soluble in a useful SCF, the GAS process may be employed; however, this process has not been applied to SLN production in the reported literature.

Owing to the nature of the lipids used in SLN production and the molecular dispersion of drug within the lipid matrix, lipid crystallization to the ß_i_ and ß-form during solidification results in a matrix with little space for incorporation of drug molecules [174,175]. To overcome this limitation, a modification of the SLN platform was developed in which the nanostructure of the solid lipid matrix was engineered to permit more space for drug molecules to be incorporated. Termed nanostructured lipid carriers (NLC), these nanoparticles retain the solid lipid matrix defining the SLN platform, but aim to create an imperfect lipid matrix, leaving more sites available for drug incorporation.

To date, few examples of SCF-mediated NLC production have been reported. In 2015, Goncalves et al. compared formulations of ketoprofen produced by a PGSS process, in which glyceryl monooleate, a liquid glycerolipid, was incorporated into the formulations of three different solid glycerolipids [176]. Although the particles generated were in the micron range, some general principles of SLN versus NLC carriers can be interpreted. Their findings demonstrate that addition of a liquid glycerolipid increased the stability of the particles compared to a pure solid formulation, with no significant change to the enthalpy of fusion after 6 months of storage for the binary systems. Encapsulation efficiency of ketoprofen was directly correlated to the mass ratio of the liquid glycerolipid, and a maximum encapsulation efficiency of 97% was achieved. These findings suggest that NLCs produced by SCF-based processes have clear advantages over other lipid formulations, but more studies and new processes may be needed to generate carriers in the sub-micron size range.

#### Section Summary

- Drug-loaded SLN preparation by SFEE, PGSS, RESS, and GAS processes has been described with a wide variety of drugs, lipids, and processing conditions.
- A key area for current and future development remains NLC production via SCF-based processes, which allow for higher drug loading capacity and improved drug release characteristics.

### 3.7. Preparation of Liposomes

Liposomes are vesicles in which an aqueous core is surrounded by one or more lipid bilayers [153]. The phospholipid hydrophobic chains are embedded in the lipid bilayer and the polar groups are exposed to the extravascular solution. Bangham et al. [177] first reported the spontaneous formation of closed bilayer structures when egg lecithin was dispersed in water, which was further confirmed by electron microscopy. Liposomes have attracted a lot of attention in recent years, since they can be used as drug carriers of water-soluble drugs in the inner aqueous core, and water-insoluble drugs in the lipid bilayers. Liposomes have been explored as drug delivery vehicles for a wide variety of molecules, including anticancer and antibacterial drugs, gene drugs, hormones, and signaling molecules [178].

Many conventional methods to prepare liposomes have been reported. Some of the most commonly used methods include the Bangham method [177], ether/ethanol injection method [179,180,181,182,183], reversed-phase evaporation technique [184], rehydration–dehydration technique [185], French press technique [186], and detergent dialysis technique [187]. Two major issues have plagued the popularity of these conventional methods as scale-up techniques: The excessive use of organic solvents, and the cumbersome/multiple preparation steps needed. Organic solvents may affect the structure of the encapsulated compound and can result in a toxic liposome. Therefore, during the preparation of drug delivery carriers, the removal of any remaining organic solvents must be ensured [188], hence necessitating the need for alternative methods, such as supercritical fluid techniques.

The first attempt at preparing liposomes using SCF CO_2_ was reported by Castor in 1994 [189]. Some of the most widely used SCF processes in liposome preparation include injection and decompression, RESS, GAS, SAS, ASES, and supercritical reverse-phase evaporation (SCRPE) methods [188,190,191]. Liposomes were also prepared in a continuous supercritical fluid process, named supercritical-assisted liposome formation (SuperLip), where controlled nanometric-sized liposomes with improved drug encapsulation efficiency were obtained [192,193]. In this process, CO_2_ and an ethanolic solution containing phospholipids were subjected to high-pressure mixing to produce an expanded liquid. The expanded liquid was then fed into a high-pressure vessel. In the same high-pressure vessel, water was continuously sprayed. The atomized water, together with the expanded liquid, led to the formation of sub-micron water droplets. The contact of the sub-micron water droplets with an ethanolic solution created an emulsion that led to the production of liposomes. Depending on the temperature and pressure of the mixer vessel, the size of the liposomes could be tuned between 130 and 294 nm. BSA was then loaded into the liposomes using BSA dissolved in water during the aqueous solution during the SCF CO_2_-based process. A high encapsulation efficiency of >85% with a liposome size of 193 nm was obtained. The study demonstrated the proof of concept for encapsulating hydrophilic drugs into liposomes using SCF CO_2_-based process.

Zhao et al. investigated the effects of concentration and types of phospholipids and sterols on the particle size, uniformity, zeta potential, and morphology of liposomes prepared using SCF CO_2_ [194]. Several other literature reports describe the use of liposomes as ideal encapsulation options to preserve drugs, anti-oxidants, vitamins [195], and dyes. Liposomes containing drugs such as Sirolimus [196], miconazole [197], soy lecithin [198], albendazole [199], and pegylated liposomes of docetaxel [200] were prepared using various SCF techniques. Campardelli et al. reported the preparation of liposomes loaded with fluorescein using an SCF-based process [201]. Fluorescein is a dye used as a marker to monitor the absorption of liposomes into tissues. To preserve anti-oxidant stability, liposomes were used as carriers [202,203,204]. Finally, there are numerous other literature reports that describe liposome formation using SCF techniques [17,195,205,206,207,208,209].

#### Section Summary

- SCF CO_2_ has been successfully employed to demonstrate the production of liposomes. The recent demonstration of continuous production of liposomes using the SuperLip process is encouraging.
- In order for widespread use of SCF CO_2_-based processes to occur, meaningful control experiments have to be done comparing SCF CO_2_-based with microfluidics-based techniques, which are now increasingly being used by researchers in the field of liposomes.

## 4. Conclusions and Perspectives

In this review, the use of SCFs in the production of API particles and drug delivery systems such as microparticles, nanoparticles, polymeric membranes, aerogels, microporous foams, solid lipid nanoparticles, and liposomes were discussed. It is clear that SCF-based methods provide unique opportunities to (1) tune size, morphology, and polymorphic form of APIs; (2) improve methods of making established drug delivery platforms such as ASDs and polymer–matrix-based carriers; (3) manufacture novel drug delivery systems such as aerogels, solid lipid nanoparticles and liposomes; and (4) tune properties of polymer membranes, which in turn show improved performance in unit operations such as filtration and dialysis. In many cases, SCF methods have shown to be superior to conventional methods in terms of tunability, higher efficiency with better controls, ability to perform continuous operations, and in being environmentally friendly. In spite of these advantages, SCF technologies are underutilized in the pharmaceutical setting, with separation-based unit operations being the exception.

It is worthwhile to consider what the gaps are in bringing these technologies to fruition:

- In the context of oral delivery, SCF-based methods have been touted as a way to reproducibly control particle size and morphology, and have been compared with conventional methods such as jet and bead milling. While SCF is indeed better than conventional technologies, more studies are needed to establish their superiority over emerging bottom-up methods such as microfluidics and flash nanoprecipitation. Secondly, from a practical perspective, there is a very high bar for introduction of new technologies in the pharmaceutical industry due to regulatory considerations. In this scenario, the use of SCF-based technology to reduce particle size and improve flow for oral delivery provides only incremental rather than transformative benefit to make the case for a shift in manufacturing technologies. However, for specialized applications such as pulmonary delivery, SCF may clearly provide the advantage over conventional milling technologies in terms of improved aerosolization, ability to combine multiple drugs, and potential ease of formulation due to reduced surface energy of the particles. Thus, choice of application areas should be carefully considered to enable commercial translation of SCF-based technologies.
- Detailed modeling efforts have been undertaken to understand phase equilibria under pressure for SCFs. However, the predictability of these models has not been good. This is especially a problem when multiple phases are involved in the SCF-operation, such as co-solvents and polymeric carriers. In these cases, extensive experimental data are needed to establish the process parameters for SCF-based methods. This has led to the belief that SCF-based technologies suffer from scale-up issues and are not reproducible and robust. Thus, there is a need for more fundamental understanding of thermodynamics and kinetics of SCF-based processes.
- Finally, SCF-based methods may have significant advantages in the manufacturing of novel drug delivery systems such as solid lipid nanoparticles and liposomes. However, very few studies have been reported in this area and more work needs to be done to understand the potential of SCFs. SCF-based methods should be compared and contrasted against appropriate controls, such as microfluidics and flash nanoprecipitation.

The issues surrounding the broader exploitation of SCF-based technologies for pharmaceutical manufacturing are likely related to the application areas and inadequate process models that enable scale-up in a material sparing fashion. A thorough investigation is warranted to understand where the gaps are between research and industrial applications. In addition, appropriate control experiments need to be done contrasting SCF techniques with newer bottom-up methods to understand their utility. If the SCF technology is brought to its full potential, it can create novel opportunities in the development of drug delivery systems.

## Appendix A

**Table A1:** Summary of recent reviews in the field.

Topics Covered in Review	Reference
A general overview of use of supercritical fluid (SCF) techniques for size control and encapsulation.	[50]
An excellent overview of the use of supercritical CO_2_-based techniques for particle size control.	[81]
Review of experimental solubility data of solids in SCFs.	[210]
A specific review on supercritical CO_2_-assisted extrusion process for foaming of biopolymers.	[161]
Review focused primarily on the use of SCF methodologies in novel drug delivery systems.	[82]
A specific review of SCF-based techniques for making drug delivery systems for cancer therapy.	[211]
Descriptions of SCF-related manufacturing processes as related to co-crystal production.	[212]
Overview of the use of SCF to process biopolymers for tissue engineering applications.	[213]
Overview of SCF-assisted impregnation process for the production of drug eluting implants.	[214]
Detailed description of SCF-based manufacturing processes with primary focus of particle size control.	[215]
